# The pro-inflammatory stimulus of zinc- and copper-containing welding fumes in whole blood assay via protein tyrosine phosphatase 1B inhibition

**DOI:** 10.1038/s41598-018-37803-0

**Published:** 2019-02-04

**Authors:** Johannes Bleidorn, Hanif Alamzad-Krabbe, Benjamin Gerhards, Thomas Kraus, Peter Brand, Julia Krabbe, Christian Martin

**Affiliations:** 1 Institute of Pharmacology and Toxicology, Medical Faculty, RWTH Aachen University, Wendlingweg 2, 52074 Aachen, Germany; 2 Institute of Occupational, Social and Environmental Medicine, Medical Faculty, RWTH Aachen University, Pauwelsstraße 30, 52074 Aachen, Germany; 3 ISF- Welding and Joining Institute, RWTH Aachen University, Pontstraße 49, 52062 Aachen, Germany

## Abstract

An asymptomatic systemic inflammation after exposure to zinc- and copper-containing welding fumes has been described as mild form of metal fume fever in recent studies. Since chronic systemic inflammation leads to a higher cardiovascular risk, examining the inflammation with the underlying pathomechanism is necessary to estimate and hopefully prevent long-term effects of welding. We established a whole blood assay to investigate the effects of zinc- and copper-containing welding fume particles on the blood immune response. Increased levels of IL-6, IL-8, TNFα and IL-1β determined after 24 hours of exposure indicated an acute systemic inflammatory reaction. *In vitro* increases of IL-6 were comparable to *in vivo* increases of serum IL-6 levels in a study with welding fume exposure of human subjects. Inhibition of PTP1B was identified as one pathway responsible for the effects of zinc- and copper-containing welding fumes and therefore welding fume fever. In conclusion, the whole blood assay is a reliable and feasible method to investigate effects of zinc- and copper-containing welding fumes on the immune system and as a surrogate for systemic inflammation and welding fume fever. Future research can utilize whole blood assays to reduce and partially replace human exposure studies for further investigations of welding fume fever.

## Introduction

Metal fume fever is a complex of symptoms including fever, headache, myalgia, fatigue and dyspnoea, typically occurring after exposure to welding fumes^1^. The symptoms persist for up to 24 hours and disappear afterwards^2^ without the need of a specific therapy^3^. Metal fume fever has mainly been described for zinc fumes, but case reports for copper also exist^1,4,5^. In studies with healthy human volunteers, a subclinical systemic inflammation indicated by an increase of C-reactive protein (CRP) serum levels could be observed 24 hours after exposure to welding fumes from a metal inert gas brazing (MIG) process containing zinc and copper^6,7^. Interestingly, the progress of the systemic inflammation seems to be in parallel with increased interleukin (IL)-6 serum levels, which can be observed as early as 6–10 hours after the beginning of the exposure^8^.

To elucidate the origin of the inflammatory response resulting in the typical symptoms shown by welders after exposure to zinc- and copper-containing welding fumes the respiratory tract including the lungs as the main uptake route of the welding fumes were investigated in prior studies. Exposure of human volunteers to zinc oxide fume resulted in increased levels of IL-6, tumor necrosis factor alpha (TNF-α) and IL-8 in broncho-alveolar lavage fluid^9^. Furthermore, approaches have been made to investigate the molecular effects of welding fumes on the lungs. *In vitro* experiments in human lung cells (A549) and rat alveolar epithelial cells indicated a zinc- and copper-mediated toxicity via generation of oxygen species^10,11^, mitochondrial dysfunction^11^, DNA-damage^12^ and induction of apoptosis^13^.

Recently, the effects of zinc- and copper-containing welding fumes have been investigated in precision-cut lung slices (PCLS)^14^. PCLS were introduced as a feasible method to investigate welding fume effects as an *ex vivo* model of the lungs. Concentration-dependent toxicity occurred in tissue slices of humans, rats and mice. Moreover, proliferation and DNA repair rates were reduced^14^. However, in contrast to the studies in human volunteers, no clear pro-inflammatory effect could be observed.

Thus, the effects of welding fumes on the lungs cannot consistently explain the observed induced systemic inflammation resulting in symptoms typical for metal fume fever.

Therefore, we investigated the effects of zinc- and copper-containing welding fumes in a whole blood assay. After incubation of whole blood with welding fumes in increasing concentrations for 24 hours the cytokine response was determined. To assess if the measured levels were similar to inhalative exposure scenarios, they were compared to serum levels determined in an exposure study with healthy volunteers^8^. Additionally, the mechanism by which zinc- and copper-containing welding fumes induce the inflammation was investigated.

## Results

### Cytokine levels in whole blood assay for zinc- and copper-containing welding fumes

**Figure 1:**
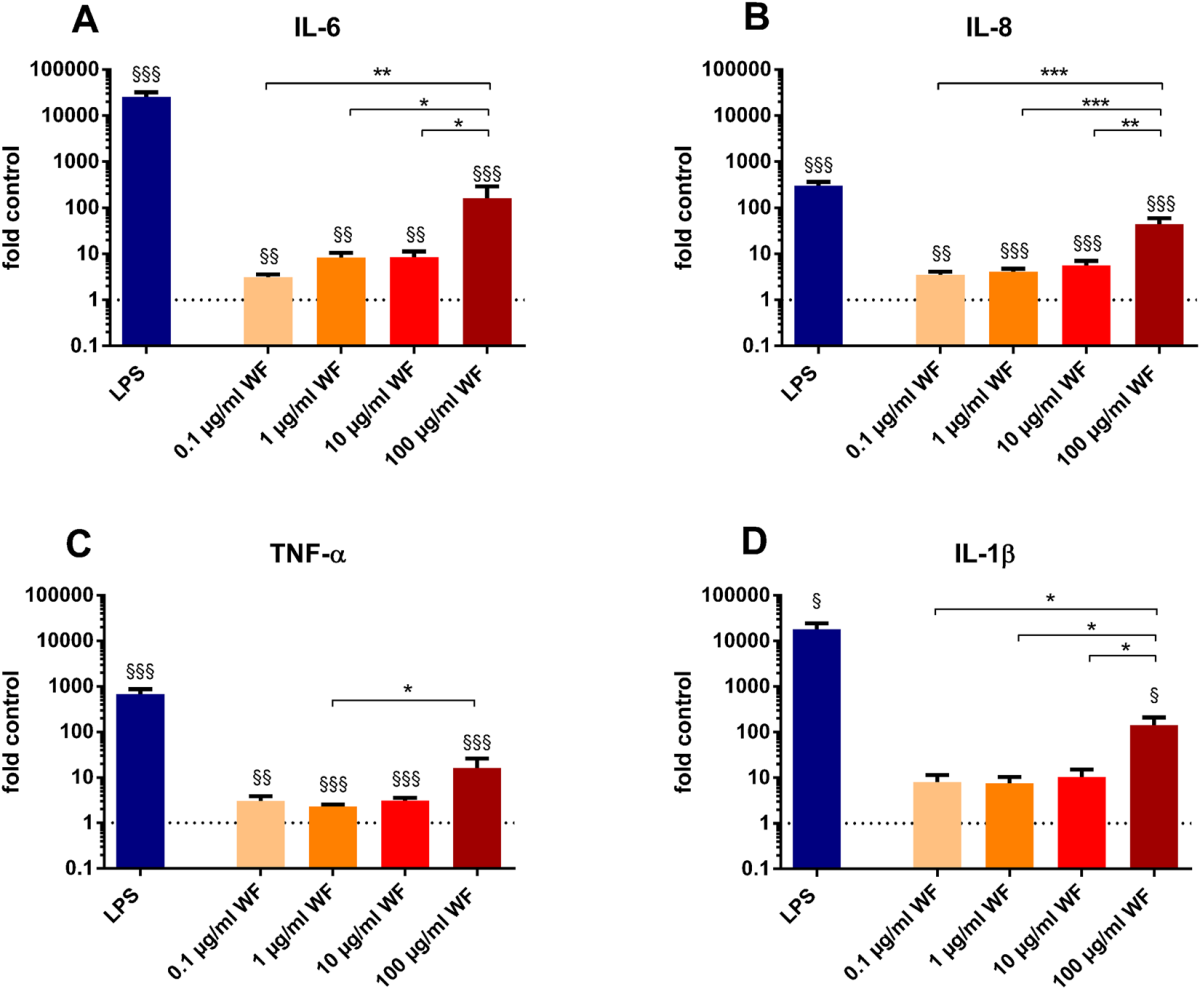
Effects of zinc- and copper-containing welding fumes on cytokine release after whole blood incubation for 24 hours with four concentrations (0.1 µg/ml, 1 µg/ml, 10 µg/ml, 100 µg/ml). Incubation with 10 ng/ml LPS was conducted as positive control (LPS). The determined levels in pg/ml were divided by the mean level of the corresponding control. The dotted line indicates the corresponding control. All data are shown as mean ± SEM, (n = 7). (**A**) IL-6 levels, (**B**) IL-8 levels, (**C**) TNF-α levels, (**D**) IL-1β. ^§^p < 0.05, ^§§^p < 0.01, ^§§§^p < 0.001 for comparisons with the control. *p < 0.05, **p < 0.01, ***p < 0.001 for comparison of differences within the welding fume concentrations.

**Figure 2:**
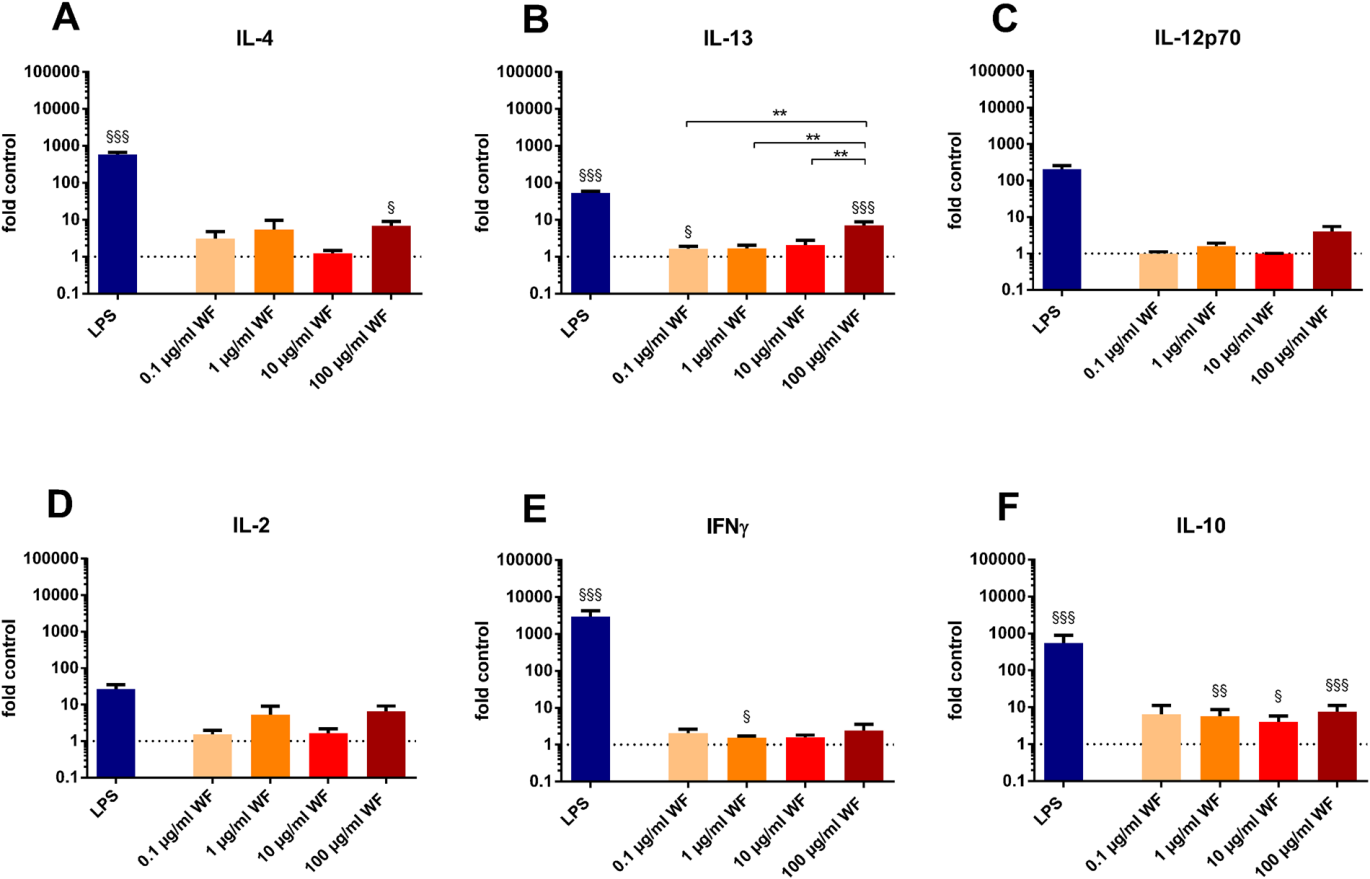
Effects of zinc- and copper-containing welding fumes on cytokine release after whole blood incubation for 24 hours with four concentrations (0.1 µg/ml, 1 µg/ml, 10 µg/ml, 100 µg/ml). Incubation with 10 ng/ml LPS was conducted as positive control (LPS). The determined levels in pg/ml were divided by the mean level of the corresponding control. The dotted line indicates the corresponding control. All data are shown as mean ± SEM, (n = 7). (**A**) IL-13 levels, (**B**) IL-10 levels, (**C**) IL-4 levels, (**D**) IFNγ levels (**E**) IL-2 levels, (**F**) IL-12p70 levels. ^§^p < 0.05, ^§§^p < 0.01, ^§§§^p < 0.001 for comparisons with the control. **p < 0.01 for comparison of differences within the welding fume concentrations.

The whole blood incubation for 24 hours with zinc- and copper-containing welding fumes with four different concentrations (0.1 µg/ml, 1 µg/ml, 10 µg/ml, 100 µg/ml) led to a significant increase of IL-6, IL-8 and TNF-α levels for all four concentrations compared to cytokine levels determined from the negative control (Fig. 1A–C). IL-1β release was significantly higher after incubation with 100 µg/ml welding fume particles (Fig. 1D). Moreover, IL-6, IL-8 and IL-1β showed significant higher levels after incubation with 100 µg/ml welding fume particles, compared to lower concentrations (Fig. 1A,B,D). After incubation with 100 µg/ml welding fume particles TNF-α levels were higher than levels after incubation with 10 µg/ml but not lower concentrations (Fig. 1C). Regarding IL-13 levels, significant increases compared to the controls were observed for 0.1 and 100 µg/ml and for the comparison of 100 µg/ml with lower concentrations (Fig. 2A). Additionally, concentrations of IL-10 were increased for incubation with 1, 10 and 100 µg/ml of welding fume compared to the controls (Fig. 2B). While IL-4 levels were increased for 100 µg/ml (Fig. 2C) and IFNγ (Fig. 2D) levels for 1 µg/ml both compared to the control levels, no significant differences were observed for other concentrations and the additionally determined cytokines IL-2 and IL-12p70 (Fig. 2E,F).

Except for IL-2 and IL-12p70, significant increased levels of all cytokines were observed following an incubation with 10 ng/ml of lipopolysaccharides (LPS) from *E. coli* (Figs 1, 2).

### Role of protein tyrosine phosphatase 1B (PTP1B) inhibition in welding fume effects

**Figure 3:**
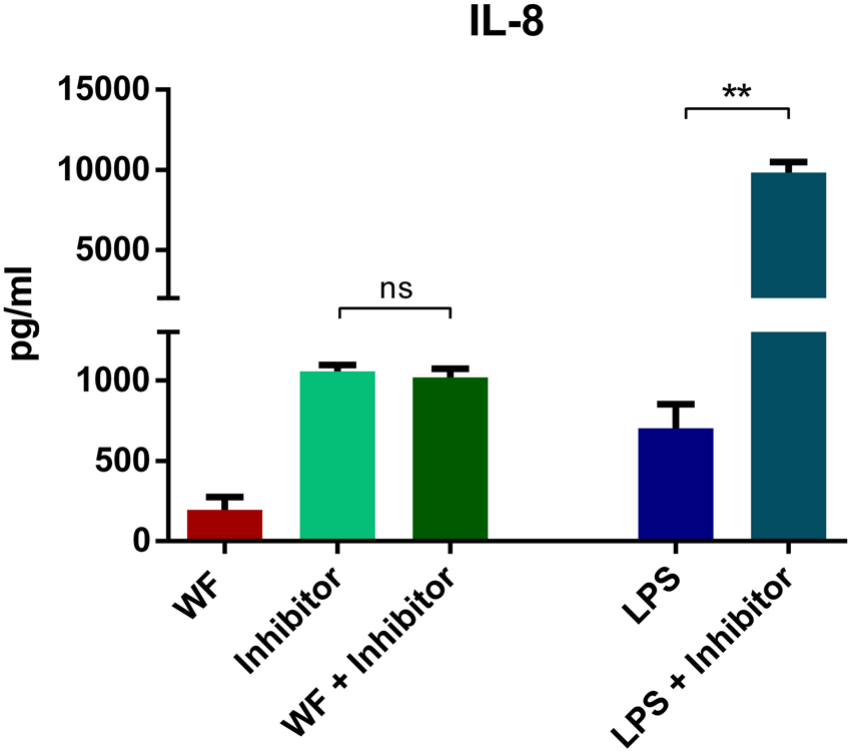
Differences in IL-8 release between welding fume incubation and PTP1B inhibition. IL-8 release after 24 hours of whole blood incubation with (1) 100 µg/ml welding fume particle concentration (WF), (2) PTP1B inhibitor 10-fold IC50 (Inhibitor), (3) combined incubation of 100 µg/ml welding fume particles and PTP1B inhibitor 10-fold IC50 (WF + Inhibitor), (4) 10 ng/ml LPS, and (5) combined incubation of PTP1B inhibitor 10-fold IC50 and 10 ng/ml LPS (LPS + Inhibitor). All data are shown as mean ± SEM, (n = 4). **p < 0.01 for comparison of differences within incubated samples, ns = not significant.

**Figure 4:**
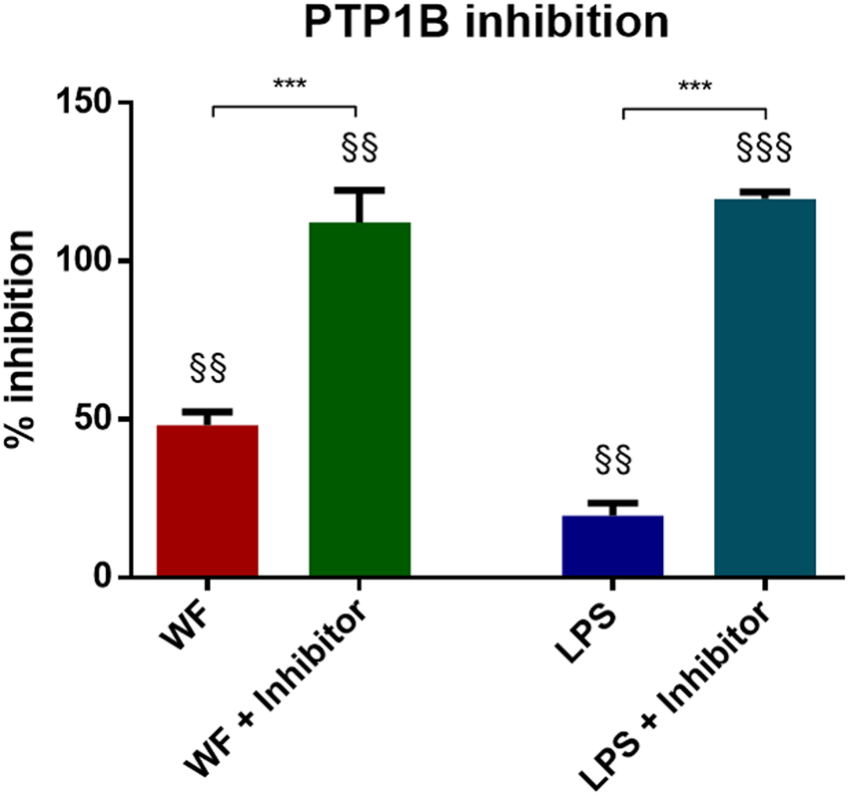
Effects of zinc- and copper-containing welding fumes on PTP1B inhibition. PTP1B inhibition was investigated after incubation with (1) 100 µg/ml zinc- and copper-containing welding fume particles (WF), (2) combined incubation of 100 µg/ml welding fume particles and PTP1B inhibitor 10-fold IC50 (WF + Inhibitor), (3) 10 ng/ml LPS, 4) combined incubation of PTP1B inhibitor 10-fold IC50 and 10 ng/ml LPS (LPS + Inhibitor). Incubation with PTP1B inhibitor 10-fold IC50 was defined as 100% inhibition and incubation with PTP1B without the presence of an inhibitor was defined as 0% inhibition. All data are shown as mean ± SEM, (n = 4–6). ^§§^p < 0.01, ^§§§^p < 0.001 for comparisons with the control (0%). ***p < 0.01 for comparison of differences within incubated samples.

Combined incubation of whole blood for 24 hours with ‘InSolution™ PTP1B Inhibitor-Calbiochem’ (PTP1B inhibitor) and 100 µg/ml of zinc- and copper-containing welding fume particles showed similar levels of IL-8 release compared to the inhibitor alone indicating no additive or synergistic effects of inhibitor and welding fumes (Fig. 3). Furthermore, incubation of the inhibitor combined with 10 ng/ml LPS resulted in a considerably increased IL-8 release compared to incubation with LPS alone indicating synergistic effects. IL-8 levels after incubation with pyrogen free water as a negative control and DMSO as a technical control DMSO were under the detection limit. Additionally, the direct PTP1B inhibition was investigated, PTP1B inhibition was significantly increased after incubation with 100 µg/ml zinc- and copper-containing welding fume particles, combined incubation of welding fume particles with the PTP1B inhibitor, 10 ng/ml LPS and LPS combined with the PTP1B inhibitor (Fig. 4). While incubation with welding fumes, as well as with LPS only achieved a partial PTP1B inhibition, the combination with the PTP1B inhibitor resulted in a considerable PTP1B inhibition.

### No LPS-meditated effects by welding fume particles

**Figure 5:**
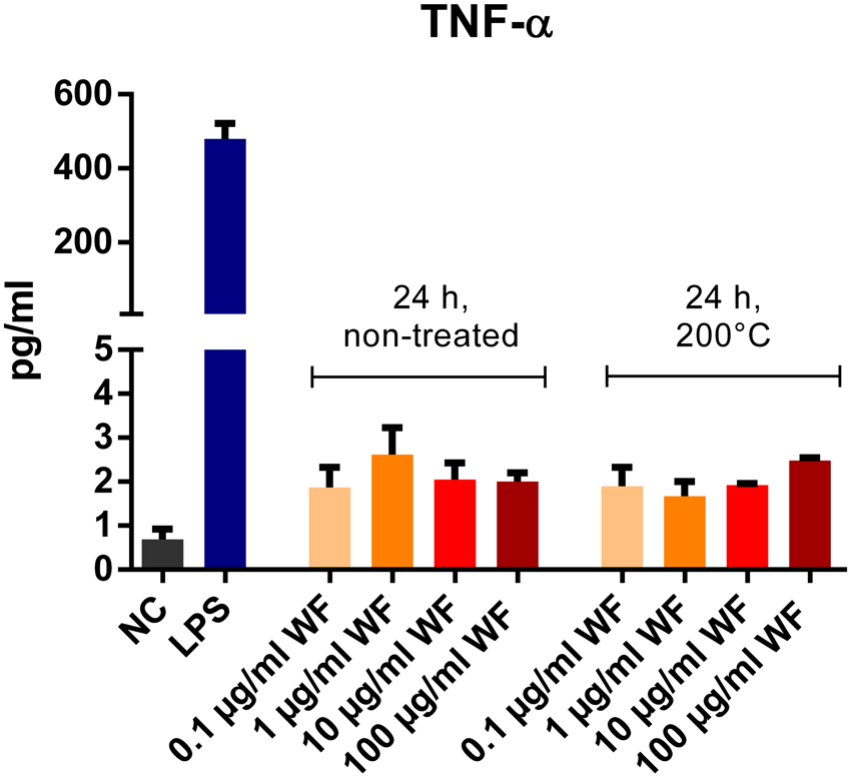
Comparison of non-treated zinc- and copper-containing welding fumes vs. welding fumes treated with 200 °C for 17 h. TNF-α release after 24 hours of incubation with increasing concentrations of welding fume particles (WF) (0.1 µg/ml, 1 µg/ml, 10 µg/ml, 100 µg/ml) was measured. Pyrogen free water was provided as negative control (NC) and 10 ng/ml LPS as positive control (LPS). Data are shown as mean ± SEM, (n = 3). Comparison of corresponding concentrations (0.1 vs. 0.1 µg/ml) for non-treated and heat-treated welding fume showed no significant differences.

Comparison of TNF-α release of whole blood incubated for 24 hours with welding fume particle concentrations either heat-treated or not, showed no significant differences, indicating an LPS-independent mechanism (Fig. 5).

### Comparison of IL-6 levels from the present study with IL-6 serum levels determined in an exposure study with 15 human volunteers

**Figure 6:**
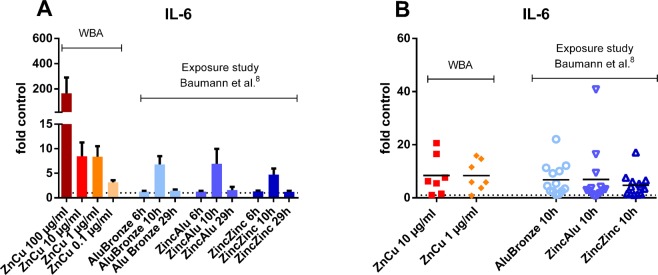
Comparison of IL-6 levels from the present study with IL-6 serum levels of healthy volunteers in an exposure study after exposure for 6 hours with the indicated welding fumes. (**A**) IL-6 levels after whole blood incubation with four different concentrations of zinc- and copper-containing welding fume particles for 24 hours and IL-6 serum levels after 6 hours of exposure to three different welding fumes containing (1) copper, zinc and aluminium (“AluBronze”), (2) zinc and aluminium (“ZincAlu”) or (3) zinc with a low amount of aluminium (1.2%) (“ZincZinc”) 6, 10 and 29 hours after the beginning of the exposure. To achieve comparability of whole blood and serum levels, the determined concentrations in pg/ml were divided by the concentration of the corresponding control. Data are shown as mean ± SEM. (n WBA = 7, n exposure study = 13). (**B**) Normalised IL-6 levels in the whole blood assay (WBA) after incubation with zinc- and copper-containing welding fumes with concentrations of 1 and 10 µg/ml compared with IL-6 serum levels determined 10 h after inhalation of the three different welding fumes described above. The determined levels in pg/ml were divided by the mean level of the corresponding control. Data are shown as mean. (n WBA = 7, n exposure study = 13). Comparison of all levels (10 and 1 µg/ml vs. 10 h AluBronze, ZincAlu and ZincZinc) showed no significant differences.

To assess if the increases of the determined whole blood cytokine levels correspond to *in vivo* scenarios, increases in IL-6 levels were compared to increases in IL-6 serum levels from an exposure study with 13 healthy male volunteers^8^ (Fig. 6A). In this study, serum levels of IL-6 were increased compared to baseline levels after 6 hours of exposure and 10 hours after the beginning of the exposure with different welding fumes containing (1) zinc, copper and aluminium, (2) zinc and aluminium or (3) zinc with a low amount of aluminium (average 1.2%)^8^. When normalised to the corresponding baseline levels, the serum levels from the exposure study correspond to the IL-6 levels of whole blood assay incubation with 10 and 1 µg/ml of welding fumes (Fig. 6B).

## Discussion

The present study introduces the whole blood assay as feasible method to investigate the effects exerted by zinc- and copper-containing welding fumes on the blood immune system. Here, the incubation of welding fume particles with whole blood resulted in a considerable increase of cytokines and chemokines IL-6, IL-8, TNF-α, IL-1β, IL-13 and IL-10. Interestingly, the increase of IL-6 was comparable to the increases of serum levels of healthy volunteers determined in a study with zinc-, copper- and aluminium-containing welding fume exposure^8^. Furthermore, as one of the underlying pathomechanisms of metal fume fever the inhibition of PTP1B by zinc- and copper-containing welding fumes could be identified.

Best to our knowledge, this is the first study to show pro-inflammatory effects of zinc- and copper-containing welding fumes on whole blood indicated by increased cytokine levels. This cytokine release can be seen as the early phase of an acute phase reaction resulting in a systemic inflammation with increased CRP and serum amyloid-A (SAA) levels in accordance to metal fume fever. IL-6, a pro-inflammatory cytokine released by circulating immune cells, fibroblasts and endothelial cells as an acute response to inflammation^15^, is able to initiate induction of the acute phase reaction in hepatocytes resulting in CRP and SAA secretion^16^. Furthermore, the release of IL-8 and TNF-α, important factors in the activation and adhesion of immune cells and initiators of inflammation cascades^17,18^, could be accountable for leukocyte chemotaxis and migration resulting in an increased amount of neutrophils in the systemic circulation^19^. Release of IL-1β, another early pro-inflammatory cytokine^20,21^, indicates involvement of the inflammasome, similarly to other inhalative exposures, e.g. cigarette smoking or diesel exhaust fumes^22,23^.

In contrary to the classic rather unspecific inflammatory cascade, a typical allergic or parasite defence reaction, indicated by the release of IL-4 and IL-13 could not be determined. Aside pro-inflammatory stimuli, IL-10 as a negative regulator of inflammation was also activated by zinc- and copper-containing welding fumes to limit the occurring inflammation.

Thus, welding fumes containing zinc and copper not only trigger an unspecific inflammation resulting in an acute phase reaction, but also activate cascades implicated in specific pathogen defence, e.g. inflammasome. If this could result in similar long-term complications as exposure to exhaust fumes and fine dust needs to be investigated. However, consecutive IL-10 secretion could represent a mechanism to prevent sustained or enhanced inflammation and limit metal fume fever.

Recently, the effects of the same welding fume particles were investigated in human PCLS^14^. Aside a concentration dependent toxicity, no clear pro-inflammatory stimulus could be identified excluding the lung cells as origin of the systemic inflammation observed in exposure of human subjects^6–8^. Thus, the induction of inflammation caused by contact of blood immune cells with welding fume particles shown in this study seems to be the origin of the inflammation resulting in a systemic inflammation and metal fume fever. While the inhalation and consecutive solution in mucus could not cause a clinical relevant amount of local toxicity^14^, the main effect of welding fumes rather occurs after either phagocytosis of welding fume particles or particle dissolution. Zinc oxide and copper oxide are mostly insoluble in water and only a small fraction of 10–15% is dissolved in cell culture medium at a pH of 7.2–7.3, representing the pH in pulmonal mucus^14^. The insoluble fraction of zinc and copper oxide can be taken up via phagocytosis into macrophages and then transferred to the phagolysosome^24^ with consecutive solution in the acid milieu^25,26^ resulting in cytotoxicity and inflammation in the corresponding cell^24^. The zinc and copper ions coming from welding particles solved in mucus could be taken directly into the blood stream exerting the effects demonstrated in this study. Moreover, deposition of agglomerates could possibly result in high local concentrations in the blood. Therefore, the comparison of results obtained in PCLS, which indicate the local tissue response and the whole blood assay on behalf of a systemic reaction allows the identification of the zinc- and copper-containing welding fume particle contact with blood immune cells as the main mechanism of the zinc- and copper-containing welding fume induced inflammation detected in welders^7,8^.

Both, zinc and copper ions^27–30^, as well as their compounds^31^ are known to inhibit PTP1B. Inhibition can be conducted directly through enzyme-binding^32,33^ or indirectly through production of reactive oxygen species with following PTP1B inhibition^34^. Due to the inhibition of PTP1B receptor tyrosine kinases (RTKs) are less dephosphorylated^35^ resulting in increased cytokine secretion^36^ and systemic inflammation^37^. PTP1B has been demonstrated to be ubiquitously expressed^38^ and to have regulatory effects in blood immune cells, such as macrophages, monocytes and granulocytes^38,39^. It has previously been described as an important negative regulator of inflammation functioning as safety mechanism preventing hyperinflammation^40^.

Although zinc- and copper-induced metal fume fever has been mentioned in case reports for the last 500 years^41–43^ and prior studies investigated the systemic inflammatory response, the pathomechanism behind the systemic inflammation was not entirely elucidated. In this study PTP1B inhibition was identified as at least a part of the mechanism responsible for the effects of zinc- and copper-containing welding fumes and metal fume fever. This is due to the observation that if welding fume particles are added to the specific inhibitor no increase in IL-8 secretion occurs. It was deduced that if the welding fume particles could cause inflammation/IL-8 secretion via another pathway than PTP1B inhibition the IL-8 level would have increased. Since the treatment with LPS and the inhibitor showed considerable higher IL-8 levels, the potential additive effect of welding fumes could have been identified if it existed. However, it is not clear if the free zinc and copper ions are responsible for the effects. Since a chelating agent was present in the PTP1B assay, at least partial effects could be caused by the agglomerated welding fume particles, e.g. zinc or copper oxides, and not the free ions.

To correlate the increases in cytokine concentrations determined in whole blood assays in this study with *in vivo* exposure scenarios corresponding to real life situations, the increases in IL- 6 levels in whole blood assays after 24 hours of incubation were compared to the increases in serum levels from an exposure study^8^ 10 hours after the beginning of a 6 hour exposure. Although in this study healthy male volunteers were exposed to “AluBronze” a combined welding fume mixture containing zinc, copper and aluminium, the pro-inflammatory effects determined after the exposure to “AluBronze” can be mainly contributed to zinc and copper, since aluminium-containing welding fumes do not induce an increase in CRP serum levels after inhalative exposure^7^. Thus, the exposure to “AluBronze” can be compared to the whole blood assay performed in our study. Furthermore exposure to zinc combined with aluminium (“ZincAlu”) as well as to welding fumes containing mainly zinc but also a small amount (1.2%) of aluminium (“ZincZinc”) showed comparable results^8^, indicating a rather zinc-driven effect. The almost identical increases in IL-6 levels compared to the corresponding controls underline the transferability of the whole blood assay to *in vivo* inhalation exposure and could enable the usage of whole blood assays as a prognostic tool to predict the occurrence of metal fume fever. Whole blood incubation is therefore feasible for screening of inflammatory effects of different welding fumes, as well as for pre-studies prior to *in vivo* exposure studies. Thus, the actual burden of inhalative exposure of human volunteers could be reduced and partially replaced. Furthermore, since human whole blood samples are easy to obtain and welding fume effects are solely relevant in human occupational scenarios, animal experiments could be replaced for certain questions following the 3R-principle of Russel and Burch^44^ requesting replacement, reduction and refinement of animal experiments.

Since even a single exposure to zinc- and copper-containing welding fumes leads to an asymptomatic systemic inflammation^6,7^, regular occupational exposure could pose a long-term risk for inflammation and following consequences. Accordingly, epidemiologic studies observed an increased risk for cardiovascular events in patients with chronic slight elevations of CRP-levels^45–47^ and welders also show an increased risk^48^. Further research needs to determine if a repeated exposure causes a repeated systemic inflammation or if counter mechanisms are activated preventing a chronic inflammation, e.g. overexpression or upregulation of PTP1B. Furthermore, PTP1B inhibition seems to pose a risk for hyperinflammation in case of a simultaneous infection, indicated by the increased PTP1B inhibition resulting in an extremely increased IL-8 release triggered by the combination of LPS and the PTP1B inhibitor. Accordingly, PTP1B has previously been described as an important negative regulator of LPS-induced inflammation in various cell types^49–51^, functioning as safety mechanism preventing hyperinflammation. If regular exposure to PTP1B inhibiting welding fumes could end in clinically relevant hyperinflammation as reaction to pulmonary or systemic infections is unclear and needs to be investigated. Furthermore, the partial inhibition of PTP1B by LPS indicates that further inflammatory pathways are involved in LPS mediated immune responses posing additional risks in infection related systemic inflammation. Other studies have shown a minor inhibition of PTP1B by LPS^52,53^ comparable to the results in this study. The inhibitions seems to be caused by LPS-mediated oxidation of the enzyme^52^. Regarding possible effects by contamination of the welding fume particles by LPS, a relevant contamination with LPS was excluded in a prior study^14^. Furthermore, equal TNF-α levels in blood samples incubated with welding fume particles with and without heat treatment proved the LPS-independent effects, since TNF-α secretion is a sensitive indicator for LPS contamination^54–56^. Thus, the pro-inflammatory effects of the zinc- and copper-containing welding fumes determined in our study can be contributed exclusively to the welding fume particles themselves.

In summary, our results demonstrate the direct pro-inflammatory effects of zinc- and copper-containing welding fumes on blood cells. We initiated the whole blood assay as a reliable and feasible method to easily assess and offer prediction of inflammatory effects of welding fume particles. The pro-inflammatory effect of zinc- and copper- containing welding fumes is caused by inhibition of the PTP1B resulting in increased activation of RTKs and consecutive cytokine release. Additionally, a risk for hyperinflammation caused by PTP1B inhibition could be possible for welding fume exposure. Further research should address, to what extent the immune response is altered in individuals with smoking habits, allergies or chronic diseases, e.g. COPD or cardiac diseases. The whole blood assay represents a good *ex vivo* method to investigating these questions providing a feasible alternative to animal experiments and human exposure studies.

## Methods

### Subjects

7 healthy male subjects were included in this study. The mean age was 27.71 ± 5.12 (SD) years. All participants were non-smokers without prior occupational welding fume exposure. The study was approved by the ethics committee of the Medical Faculty Aachen, RWTH Aachen University (EK 262/17) and was performed in compliance with the Declaration of Helsinki ethical principles for medical research involving human subjects. All subjects gave written informed consent prior to inclusion.

### Particle production and characterization

The production and characterisation of the welding fume particle has been described before^14^. In brief, the welding fume was produced using metal-inert-gas (MIG) brazing of hot-dip galvanised steel using a low alloy copper wire and an impulse arc process. Welding was performed under a sampling hood; the fume was collected on an ashless paper filter and the particles were removed using a small brush. The median particle diameter was 120 nm. Particles were polydisperse with a geometric standard deviation of 1.6. The welding fume samples used in this study contained 53% zinc and 24% copper using Atomic Absorption Spectrometry techniques^14^.

### Agents

RPMI-1640 Medium, modified with 20 mM HEPES and L-glutamine, was purchased from Sigma-Aldrich (Steinheim, Germany). Pyrogen-free water and ‘InSolution™ PTP1B Inhibitor-Calbiochem’ were purchased from Merck (Darmstadt, Germany). PICO50 arterial blood sampler preheparinized with 80IU dry electrolyte-balanced heparin were purchased from Radiometer (Krefeld, Germany). The PTP1B Colorimetric Assay Kit was from BPSBioscience (San Diego, CA, USA). Lipopolysaccharide (Escherichia coli O111.B4), was purchased from Sigma-Aldrich (Steinheim, Germany).

### Whole blood assay

7 healthy male subjects donated 10 ml blood. It was collected from each individual into five preheparinized 2.0 ml PICO50 arterial blood samplers each containing an 80IU dry heparin tablet. To account for possible circadian differences, the blood collecting was performed between 1 and 2 p.m. for each subject. After gentle mixing of blood and heparin via inverting and rolling of the syringes, the blood was transferred in 2 ml Eppendorf reaction vessels each containing 1200 µl RPMI and additionally either 300 µl of pyrogen-free water for the negative control, 300 µl of 10 ng/ml LPS for the positive control or a solution of 300 µl of zinc- and copper-containing welding fume particles in RPMI resulting in four different concentrations (0.1 µg/ml, 1 µg/ml, 10 µg/ml, 100 µg/ml) with an overall volume of 1800 µl in each vessel. The reaction vessels were put in an incubator with humid atmosphere at 37 °C. After 24 hours of incubation samples were centrifuged at 10.000 × *g* for 5 minutes and supernatants were aliquoted and immediately frozen. Samples were stored at −80 °C until further investigations.

### PTP1B inhibition

For the investigation of the pathomechanism causing the inflammation, blood samples of 4 subject were also incubated with PTP1Binhibitor. 300 µl of the blood was incubated with 1200 µl RPMI and 300 µl of agent solution diluted in RPMI. Samples with the following treatments were incubated: (1) 100 µg/ml zinc- and copper-containing welding fume particles, (2) 40 µM of ‘InSolution™ PTP1B Inhibitor-Calbiochem’ solved in DMSO (0.4%) for an effective dose of 10-fold IC50. (3) 100 µg/ml welding fume particles combined with 40 µM PTP1Binhibitor, (4) 10 ng/ml LPS (5) 10 ng/ml LPS and 40 µM PTP1B inhibitor. Additionally, a technical control containing 0.4% DMSO in RPMI was included.

### PTP1B inhibition assay

For the investigation of PTP1B inhibition a PTP1B Colometric Assay Kit was performed according to the manufacturer’s instructions (BPSBioscience, San Diego, CA, USA) including dithiothreitol (DTT) addition. PTP1B inhibition was examined after incubation with (1) 100 µg/ml zinc- and copper-containing welding fume particles, (2) 100 µg/ml welding fume particles combined with 40 µM of PTP1B inhibitor, (3) 10 ng/ml LPS, (4) 10 ng/ml LPS and 40 µM PTP1B inhibitor. To calculate the relative PTP1B inhibition of those 4 treatments, a positive control indicating 0% enzyme inhibition and the PTP1B inhibitor indicating 100% enzyme inhibition were included.

### Exclusion of LPS-meditated effects of welding fume particles

The LPS content of the welding fume has been determined in a prior study and levels were under the biological effective limit^14^. TNF-α release after 24 hours of incubation was determined in blood samples donated by 3 volunteers with welding fume particles, which underwent heat-treatment at 200 °C for 17 hours before usage to guarantee LPS inactivation. Incubation with both welding fumes, either heat treated or not, was performed with 4 different welding fume particle concentrations (0.1 µg/ml, 1 µg/ml, 10 µg/ml, 100 µg/ml).

### Cytokine levels

Levels of IL-1β, IL-2, IL-4, IL-6, IL-8, IL10, IL-12p70, IL-13, TNF-α and IFNγ were analysed via an electrochemiluminescent immunoassay (V-PLEX Proinflammatory Panel 1 Human Kit) according to the manufacturer’s instructions (Meso Scale Discovery (MSD), Gaithersburg, MD, USA) using the Meso Quick Plex SQ 120 (Figs 1, 2 and 4–6). Raw data were analysed using the Discovery Workbench 4.0 software (MSD). For one experiment IL-8 levels were determined by a commercially available enzyme-linked immunosorbent assay (ELISA) according to the manufacturer’s instructions (R&D Systems, Inc., Minneapolis, MN, USA) (Fig. 3).

### Comparison to inhalative exposure study

To assess comparability of whole blood assay and *in vivo* exposure, increases in IL-6 levels determined in whole blood assay were compared to increases in IL-6 serum levels from an exposure study with 13 healthy male volunteers conducted in the Aachen workplace simulation laboratory^8^. On 4 different exposure days, the volunteers were exposed to ambient air or three different welding fumes for 6 h. The welding fumes were gained from different welding processes including (1) brazing of galvanized steel using an aluminium bronze wire (AluBronze), (2) joining of galvanized steel and aluminium using a zinc wire (ZincAlu) and (3) brazing of galvanized steel using a zinc wire (ZincZinc). Subjects were exposed to an average particle mass concentration of 2,5 mg/m^3^ Alubronze, 2.0 mg/m^3^ ZincAlu or 2.0 mg/m^3^ ZincZinc, respectively. Serum samples were collected before and 6, 10 and 29 hours after the beginning of the exposure.

### Statistics

Data analysis was performed using GraphPad Prism 6 (GraphPad, La Jolla, USA) and JMP 13.1.0 (SAS Institute Inc., Cary, North Carolina, USA). All data are shown as mean ± SEM except in Fig. 6B (mean only) and n indicates the number of blood donors.

Data were tested for normal distribution using Shapiro Wilk test and Brown Forsythe test was used to check for equal variances. If suitable, Box Cox transformation was performed to achieve normal distribution and homoscedasticity.

To test for differences of each variable from the control, one-sample t-tests against the transformed control values were performed (GraphPad, La Jolla, USA) and corrected for multiple comparisons by false discovery rate (FDR).

To assess differences between groups, one-way analysis of variances (ANOVA) was performed followed by Tukey’s multiple comparisons test (GraphPad, La Jolla, USA).

If normal distribution could not be achieved by transformation, the Wilcoxon signed-rank test was performed to test for differences between variables and control. For determination of differences between the welding fume concentrations, the Kruskal-Wallis test followed by Dunn’s multiple comparisons test was used (GraphPad, La Jolla, USA).

Analysis differences were assumed to be significant with p < 0.05.

## Data Availability

The datasets generated during and/or analysed during the current study are available from the corresponding author on reasonable request.

## References

[1] Kaye P, Young H, O’Sullivan I (2002). Metal fume fever: a case report and review of the literature. Emerg. Med. J..

[2] Ahsan SA, Lackovic M, Katner A, Palermo C (2009). Metal fume fever: a review of the literature and cases reported to the Louisiana Poison Control Center. J. La. State Med. Soc. Off. Organ La. State Med. Soc..

[3] Greenberg MI, Vearrier D (2015). Metal fume fever and polymer fume fever. Clin. Toxicol. Phila. Pa.

[4] Wong A, Greene S, Robinson J (2012). Metal fume fever - a case review of calls made to the Victorian Poisons Information Centre. Aust. Fam. Physician.

[5] Borak J, Cohen H, Hethmon TA (2000). Copper exposure and metal fume fever: lack of evidence for a causal relationship. AIHAJ J. Sci. Occup. Environ. Health Saf..

[6] Markert A (2016). Single and Combined Exposure to Zinc- and Copper-Containing Welding Fumes Lead to Asymptomatic Systemic Inflammation. J. Occup. Environ. Med..

[7] Hartmann L (2014). Assessment of the biological effects of welding fumes emitted from metal inert gas welding processes of aluminium and zinc-plated materials in humans. Int. J. Hyg. Environ. Health.

[8] Baumann R (2016). IL-6, a central acute-phase mediator, as an early biomarker for exposure to zinc-based metal fumes. Toxicology.

[9] Blanc PD, Boushey HA, Wong H, Wintermeyer SF, Bernstein MS (1993). Cytokines in metal fume fever. Am. Rev. Respir. Dis..

[10] Ahmad J (2016). Differential cytotoxicity of copper ferrite nanoparticles in different human cells. J. Appl. Toxicol. JAT.

[11] Kim YH (2010). Alveolar Epithelial Cell Injury Due to Zinc Oxide Nanoparticle Exposure. Am. J. Respir. Crit. Care Med..

[12] Akhtar MJ (2016). Dose-dependent genotoxicity of copper oxide nanoparticles stimulated by reactive oxygen species in human lung epithelial cells. Toxicol. Ind. Health.

[13] Arnal N, de Alaniz MJT, Marra CA (2013). Effect of copper overload on the survival of HepG2 and A-549 human-derived cells. Hum. Exp. Toxicol..

[14] Krabbe J (2018). The effects of zinc- and copper-containing welding fumes on murine, rat and human precision-cut lung slices. J. Trace Elem. Med. Biol..

[15] Kishimoto T (1989). The biology of interleukin-6. Blood.

[16] Tanaka T, Kishimoto T (2014). The biology and medical implications of interleukin-6. Cancer Immunol. Res..

[17] Bickel M (1993). The role of interleukin-8 in inflammation and mechanisms of regulation. J. Periodontol..

[18] Bradley JR (2008). TNF-mediated inflammatory disease. J. Pathol..

[19] Dierschke K (2017). Acute respiratory effects and biomarkers of inflammation due to welding-derived nanoparticle aggregates. Int. Arch. Occup. Environ. Health.

[20] Oettgen, H. & Broide, D. H. 1 - Introduction to mechanisms of allergic disease. In *Allergy (Fourth Edition)* 1–32, 10.1016/B978-0-7234-3658-4.00005-6 (Saunders, W. B. 2012).

[21] Gruys E, Toussaint MJM, Niewold TA, Koopmans SJ (2005). Acute phase reaction and acute phase proteins. J. Zhejiang Univ. Sci. B.

[22] Colarusso C (2017). Effect of ultrafine particle matter on human peripheral blood mononuclear cells of chronic obstructive pulmonary disease patients: involvement of the inflammasome. Eur. Respir. J..

[23] Ather JL, Martin RA, Ckless K, Poynter ME (2014). Inflammasome Activity in Non-Microbial LungInflammation. J. Environ. Immunol. Toxicol..

[24] Xia T (2008). Comparison of the mechanism of toxicity of zinc oxide and cerium oxide nanoparticles based on dissolution and oxidative stress properties. ACS Nano.

[25] Cho W-S (2011). Progressive severe lung injury by zinc oxide nanoparticles; the role of Zn2+ dissolution inside lysosomes. Part. Fibre Toxicol..

[26] Studer AM (2010). Nanoparticle cytotoxicity depends on intracellular solubility: comparison of stabilized copper metal and degradable copper oxide nanoparticles. Toxicol. Lett..

[27] Wang Q (2010). Potent inhibition of protein tyrosine phosphatase 1B by copper complexes: implications for copper toxicity in biological systems. Chem. Commun. Camb. Engl..

[28] Lu L, Zhu M (2011). Metal-based inhibitors of protein tyrosine phosphatases. Anticancer Agents Med. Chem..

[29] Wilson M, Hogstrand C, Maret W (2012). Picomolar concentrations of free zinc(II) ions regulate receptor protein-tyrosine phosphatase β activity. J. Biol. Chem..

[30] Haase H, Maret W (2003). Intracellular zinc fluctuations modulate protein tyrosine phosphatase activity in insulin/insulin-like growth factor-1 signaling. Exp. Cell Res..

[31] Samet JM, Silbajoris R, Wu W, Graves LM (1999). Tyrosine phosphatases as targets in metal-induced signaling in human airway epithelial cells. Am. J. Respir. Cell Mol. Biol..

[32] Bellomo E, Birla Singh K, Massarotti A, Hogstrand C, Maret W (2016). The metal face of protein tyrosine phosphatase 1B. Coord. Chem. Rev..

[33] Bellomo E, Abro A, Hogstrand C, Maret W, Domene C (2018). Role of Zinc and Magnesium Ions in the Modulation of Phosphoryl Transfer in Protein Tyrosine Phosphatase 1B. J. Am. Chem. Soc..

[34] Tsai C-Y, Finley JC, Ali SS, Patel HH, Howell SB (2012). Copper influx transporter 1 is required for FGF, PDGF and EGF-induced MAPK signaling. Biochem. Pharmacol..

[35] Stuible M, Tremblay ML (2010). In control at the ER: PTP1B and the down-regulation of RTKs by dephosphorylation and endocytosis. Trends Cell Biol..

[36] Nasimian A, Taheripak G, Gorgani-Firuzjaee S, Sadeghi A, Meshkani R (2013). Protein tyrosine phosphatase 1B (PTP1B) modulates palmitate-induced cytokine production in macrophage cells. Inflamm. Res. Off. J. Eur. Histamine Res. Soc. Al.

[37] Heinonen KM (2004). T-cell protein tyrosine phosphatase deletion results in progressive systemic inflammatory disease. Blood.

[38] Través PG (2014). Pivotal role of protein tyrosine phosphatase 1B (PTP1B) in the macrophage response to pro-inflammatory and anti-inflammatory challenge. Cell Death Dis..

[39] Pao LI, Badour K, Siminovitch KA, Neel BG (2007). Nonreceptor protein-tyrosine phosphatases in immune cell signaling. Annu. Rev. Immunol..

[40] Berdnikovs S (2012). PTP1B deficiency exacerbates inflammation and accelerates leukocyte trafficking *in vivo*. J. Immunol. Baltim. Md 1950.

[41] Teleky, L. Zink- und Kupferfieber. In 103–106, 10.1007/978-3-642-86862-7_5 (1955).

[42] M.D RAK (1948). Metal Fume Fever. Am. Ind. Hyg. Assoc. Q..

[43] Ross DS (1974). Welders’ Metal Fume Fever. Occup. Med..

[44] Russell, W. M. S. & Burch, R. L. The Principles of Humane Experimental Technique. *Johns Hopkins Bloomberg School of Public Health* Available at, http://altweb.jhsph.edu/pubs/books/humane_exp/het-toc.

[45] Emerging Risk FC (2012). C-reactive protein, fibrinogen, and cardiovascular disease prediction. N. Engl. J. Med..

[46] Cushman M (2005). C-Reactive Protein and the 10-Year Incidence of Coronary Heart Disease in Older Men and Women: The Cardiovascular Health Study. Circulation.

[47] Clearfield MB (2005). C-reactive protein: a new risk assessment tool for cardiovascular disease. J. Am. Osteopath. Assoc..

[48] Ibfelt E, Bonde JP, Hansen J (2010). Exposure to metal welding fume particles and risk for cardiovascular disease in Denmark: a prospective cohort study. Occup. Environ. Med..

[49] Borges B (2015). Protein tyrosine phosphatase-1B contributes to LPS-induced leptin resistance in male rats. Am. J. Physiol. Endocrinol. Metab..

[50] Song GJ (2016). A novel role for protein tyrosine phosphatase 1B as a positive regulator of neuroinflammation. J. Neuroinflammation.

[51] Xu H (2008). Phosphatase PTP1B negatively regulates MyD88- and TRIF-dependent proinflammatory cytokine and type I interferon production in TLR-triggered macrophages. Mol. Immunol..

[52] Grinnell KL, Chichger H, Braza J, Duong H, Harrington EO (2012). Protection against LPS-induced pulmonary edema through the attenuation of protein tyrosine phosphatase-1B oxidation. Am. J. Respir. Cell Mol. Biol..

[53] Aydemir TB, Sitren HS, Cousins RJ (2012). The Zinc Transporter Zip14 Influences c-Met Phosphorylation and Hepatocyte Proliferation During Liver Regeneration in Mice. Gastroenterology.

[54] Gao B, Wang Y, Tsan M-F (2006). The heat sensitivity of cytokine-inducing effect of lipopolysaccharide. J. Leukoc. Biol..

[55] Gao B, Tsan M-F (2003). Endotoxin contamination in recombinant human heat shock protein 70 (Hsp70) preparation is responsible for the induction of tumor necrosis factor alpha release by murine macrophages. J. Biol. Chem..

[56] Kirikae T (1997). Endotoxin contamination in fetal bovine serum and its influence on tumor necrosis factor production by macrophage-like cells J774.1 cultured in the presence of the serum. Int. J. Immunopharmacol..

